# The clinical safety of generic and brand clopidogrel in patients undergoing carotid endarterectomy: a randomized controlled trial

**DOI:** 10.1097/MS9.0000000000000827

**Published:** 2023-05-17

**Authors:** Fahmi H. Kakamad, Dana H. Baqi, Marwan N. Hassan, Karzan M. Salih, Hawbash M. Rahim, Shvan H. Mohammed, Berun A. Abdalla, Rawezh Q. Salih, Rebwar A. Ali, Suhaib H. Kakamad, Hiwa O. Abdullah

**Affiliations:** a College of Medicine, University of Sulaimani, Sulaimani; b Smart Health Tower, Madam Mitterrand Str; c Kscien Organization for Scientific Research, Hamdi Str. Slemani; d Medical Laboratory Science Department, Komar University of Science and Technology, Sulaymaniyah, Kurdistan, Iraq

**Keywords:** internal carotid artery stenosis, endarterectomy, antiplatelet, haematoma, stroke

## Abstract

Although some recent studies have found that original and generic clopidogrel brands are acceptable and have the same pharmacokinetic and pharmacodynamic properties, there is insufficient evidence to compare the clinical effectiveness and safety of brand and generic clopidogrel. The current study aims to evaluate the clinical safety of brand and generic clopidogrel by comparing clinical outcomes in patients undergoing carotid endarterectomy (CEA).

**Method:**

This was a single centre, parallel-arm, phase III, open-label, and randomized group sequential trial. It was conducted to compare the clinical safety of a brand and three generic clopidogrel forms in patients who have undergone CEA. All enrolled subjects were treated perioperatively with dual antiplatelet (aspirin and clopidogrel). The involved participants were assigned randomly into four groups based on the type of clopidogrel. Safety parameters were measured, including haematoma, blood draining from drainage, mouth deviation, tongue deviation, and stroke. SPSS software was used to perform the data analysis.

**Results:**

The trial included 80 patients in total (20 patients per group). Thirty-one (38.8%) patients were male. The mean age of patients was 65.6 years (49–79). Eighteen (22.5%) patients had a history of previous coronary intervention, and seventeen (21.3%) had symptomatic carotid artery stenosis. Overall, Plavix or Piax combined with aspirin were linked to better clinical safety than the other two generic clopidogrel, as the amount of bleeding was nearly two times lower in patients treated with Plavix or Piax (270±92.39 and 271.5±80.60, respectively) compared to PlavigrelAwa or Plavineer (505.7±169.1 and 496.5±174.6, respectively) (*P*≤0.001).

**Conclusion:**

The findings of the current study showed diversity in clinical safety of different clopidogrel formulations that were provided perioperatively in CEA patients.

## Introduction

Highlights

- Carotid endarterectomy (CEA) is a well-known surgical procedure used to avoid stroke in individuals with substantial carotid artery stenosis, both symptomatic and asymptomatic.
- The current study aimed to evaluate the clinical safety of brand and generic clopidogrel by comparing clinical outcomes (complications) in patients undergoing CEA.
- The combination of aspirin and brand clopidogrel (Plavix) and generic clopidogrel (Piax) is linked with greater clinical safety than other generic clopidogrel forms.
- The current study’s findings revealed differences in the clinical safety of four different clopidogrel products administered perioperatively in patients undergoing CEA.

Carotid endarterectomy (CEA) is a well-known surgical procedure used to avoid stroke in individuals with substantial carotid artery stenosis, both symptomatic and asymptomatic^1^. Most recent studies have revealed that the combination of clopidogrel and aspirin is effective in a variety of vascular operations^2^. Following the initial stroke, early antiplatelet treatment with CEA appears to be highly effective in lowering the risk of thromboembolism and the likelihood of restenosis^3,4^. Stroke is the most apparent consequence of CEA and the specific outcome that the treatment is designed to avoid^5^. Moreover, postoperative neck haematoma is a typical but possibly fatal consequence of CEA that can have catastrophic complications, such as cardiorespiratory failure and is also one of the leading causes of surgical re-exploration after this operation^6^. Surgeons are commonly faced with the decision of whether or not to continue clopidogrel medication during CEA. Some prefer to stop taking clopidogrel because they assume it increases the risk of haemorrhagic consequences^7^. Others believe that if clopidogrel is stopped, there is an increased risk of perioperative thrombotic problems, including stroke or myocardial infarction^8^. A recent study found that surgeons would discontinue clopidogrel before CEA in 55% of asymptomatic and 43% of symptomatic patients^7^. Combined antiplatelet therapy appeared to result in haematoma with progressively severe outcomes, which is becoming a more common CEA complication^5^. Clopidogrel’s high cost has been highlighted as a factor in the early termination of antiplatelet medication, resulting in thrombotic events in that period. As a result, alternative clopidogrel formulations and generics are employed to reduce expenses^9^. Although some recent studies have found that brand and generic clopidogrel are acceptable and have the same pharmacokinetic and pharmacodynamic properties, there is insufficient evidence to compare the clinical effectiveness and safety of brand and generic clopidogrel^9^. As a result, the existing evidence is limited in this regard^10^.

The current study aimed to evaluate the clinical safety of brand and generic clopidogrel by comparing clinical outcomes (complications) in patients undergoing CEA.

## Method

### Study design and setting

The current study is a single centre, parallel-arm, phase III, open-label, randomized group sequential trial. The trial was conducted in line with CONSORT criteria^11^ to compare the clinical safety of brand and three generic forms of clopidogrel in patients who have undergone CEA in the department of cardiovascular surgery at SMART Health Tower. The study was carried out in accordance with Good Clinical Practice standards and the principles outlined in the most popular version of the Helsinki Declaration. Ethical approval was obtained from the ethical committee of the University of Sulaimani, and written informed consent was obtained from each patient before the initiation of any intervention. The study details have been registered in the Research Registry (No. researchregistry8830). https://www.researchregistry.com/register-now#home/registrationdetails/6433dd0f22d8c800278faa43/

### Participants

**Figure 1:**
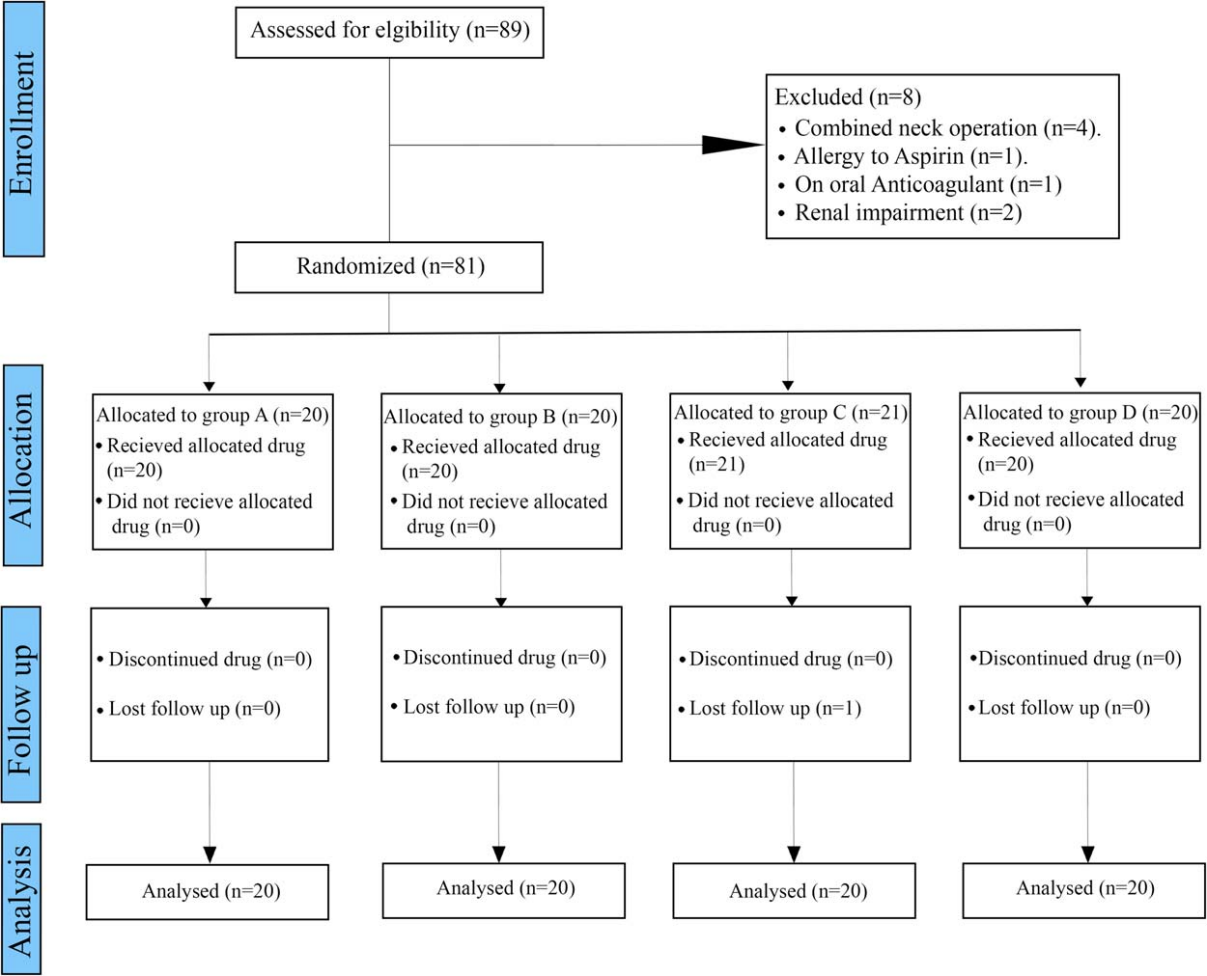
The CONSORT flow chart of patient enrolment and inclusion.

The study participants included patients who had internal carotid artery stenosis and had undergone CEA between January 2020 and June 2022. Subjects were excluded from the study if they met any of the following criteria: (1) renal impairment; (2) inability to receive aspirin for any reason (gastrointestinal problem, allergy); (3) bleeding disorder; (4) previous anticoagulant use; or (5) concurrent neck surgery. The CONSORT flow chart of patient enrolment and inclusion is shown in Fig. 1.

### Randomization and masking

Following the confirmation of eligibility, the participants’ computerized files were first admitted to a designated inbox in Smart Health Tower’s database. The second registration was completed after verifying all preoperative parameters for inclusion by computerized assignment. Member of the research team randomly generated allocation sequence codes (1:1 allocation ratio) by using SAS statistical software and simple random sampling to randomly assign the patients into four groups. Group A received (PlavigrelAwa + Aspirin), group B (Plavineer + Aspirin), group C (Piax + Aspirin), and group D (Plavix + Aspirin).

### Treatment protocol

All enrolled participants perioperatively received aspirin (100 mg of the same product) and clopidogrel (75 mg) orally. For each group, the medication started 7 days before the operation and continued postoperatively. The dose has been chosen in accordance with local standards of practice and prescribing instructions for the chosen medication. The four groups were given four different products of clopidogrel in a double-blind fashion. Neither the patient nor the surgeon knew which product was given to them.

### Intervention

Measurement was done according to the European Carotid Surgery Trial (ECST)^12^. All operations were done under general anaesthesia by the same surgeon. Patch and shunting were not used.

### Postoperative care and outcome measures

Patients were followed up on a regular basis for one month after surgery. Each patient was assessed for postoperative complications (safety parameters), including haematoma, blood draining from drainage (bleeding), mouth deviation, tongue deviation, and stroke. A small haematoma was a haematoma that developed around a wound. A medium-sized haematoma was one that extended to the neck but not to the chest. A large haematoma involving the chest. There were no persistent neurological deficits, deaths, reoperations, or postoperative strokes throughout the follow-up period.

### Statistical analysis

The collected data were analyzed using the Statistical Package for the Social Sciences (SPSS) 25.0 software. The qualitative data were presented as frequencies and percentages, and they were compared using the χ^2^ test (Fisher’s exact test was used when at least 20% of the cell counts were lower than 5). Quantitative data were analyzed using a one-way ANOVA test, and the data were presented with averages and standard deviations; if the result was found to be significant, a Bonferroni post hoc test was performed to adjust the *P* value. The purpose of applying the Bonferroni correction was to decrease the likelihood of obtaining false-positive outcomes (type I errors) when conducting multiple pairwise tests on a single dataset. A *P* value of less than 0.05 was considered significant.

### Sample size and power calculations

For trials with continuous data, a minimum of 35 patients per each group (a total of 140 patients) is required to reach statistical power of 95% at a significance level of 0.05. However, since this was an initial trial, a formal power calculation could not be performed. but, based on our preliminary experience, we estimated that enroling a total of 80 patients would be suitable for this pilot study.

## Results

The trial included 80 patients in total. They were randomly allocated into four groups of twenty patients each. Overall, the mean age of patients was 65.6 years, ranging from 49 to 79 years. Thirty-one (38.8%) of the patients were male. Eighteen patients were smokers. Hypertension was present in 65 (81.25%) patients and diabetes mellitus in 59 (73.75%) patients. Eighteen (22.5%) patients has previous coronary intervention. Symptomatic carotid artery stenosis was present in 17 (21.3%) patients. The mean duration of operation was 72.88 min, ranging from 55 to 95 min. Table 1 shows the baseline characteristics of the participants and the distribution of each group.

**Table 1:** The baseline characteristic of the participants and distribution of each group

	Groups, *n* (%)				
Variable	A	B	C	D	*P*
Age (years), Mean±SD	67.60±8.34	63.25±7.56	67.50±9.50	64.05±8.91	0.251
Sex					
Male	8 (40)	6 (30)	10 (50)	7 (35)	0.606
Female	12 (60)	14 (70)	10 (50)	13 (65)	
Smoking					
No	15 (75)	15 (75)	16 (80)	16 (80)	1.000
Yes	5 (25)	5 (25)	4 (20)	4 (20)	
HTN					
No	2 (10)	4 (20)	4 (20)	5 (25)	0.745
Yes	18 (90)	16 (80)	16 (80)	15 (75)	
DM					
No	6 (30)	5 (25)	6 (30)	4 (20)	0.871
Yes	14 (70)	15 (75)	14 (70)	16 (80)	
Previous coronary intervention					
No	14 (70)	17 (85)	16 (80)	15 (75)	0.807
Yes	6 (30)	3 (15)	4 (20)	5 (25)	
Symptomatic stenosis					
No	15 (75)	17 (85)	16 (80)	15 (75)	0.931
Yes	5 (25)	3 (15)	4 (20)	5 (25)	
Type of symptomatic stenosis:					
TIA	2 (40)	2 (66.7)	2 (50)	2 (50)	1.000
Stroke	2 (40)	1 (33.3)	1 (25)	1 (25)	
Amaurosis fugas	1 (20)	0 (0)	1 (25)	1 (25)	
Duration of operation (min), Mean±SD	73.50±9.19	70±7.94	73±10.43	75±9.17	0.383

DM, diabetes mellitus; HTN, hypertension; TIA, Transient ischemic attack.

A medium-sized haematoma was developed in 25 (31.3%) patients, but a large-sized haematoma developed in 55 (68.8%) patients. Regardless of size, the development of haematoma did not significantly differ among the group. All patients were managed conservatively and did not require reoperation. The mean amount of bleeding of all the patients was 386 ml, and a one-way ANOVA revealed that there was a statistically significant difference in the mean amount of bleeding between the groups [F(3, 76) = (19.110), *P*<0.001]. Bonferroni post hoc test for multiple comparisons found that the mean value of bleeding amount was significantly different between Group A and Group D and C (*P*<0.001) and between Group B and Group D and C (*P*<0.001). However, there was no statistically significant difference in the mean amount of bleeding between Group A and B (*P*=1.000, 505.7±169.1 and 496.5±174.6, respectively) or between Group C and D (*P*=1.000, 270±92.39 and 271.5±80.60, respectively). Mouth deviation developed in 16 (20%) patients, and nearly two-thirds were in groups A and B (5 and 6, respectively); however, the difference was still statistically insignificant (*P*=0.373). Overall, tongue deviation occurred in seven (8.8%) patients, with three cases being in group A, two in group B, and one in each of groups C and D. The complications associated with each group are shown in Table 2.

**Table 2:** The complication rate of each group

	Groups, *N* (%)				
Variables	A	B	C	D	*P*
Haematoma					0.887
Medium	5 (25)	6 (30)	7 (35)	7 (35)	
Large	15 (75)	14 (70)	13 (65)	13 (65)	
Amount of bleeding (ml), Mean±SD	505.7±169.1	496.5±174.6	270±92.39	271.5±80.60	<0.001
Mouth deviation					0.441
No	15 (75)	14 (70)	18 (90)	17 (85)	
Yes	5 (25)	6 (30)	2 (10)	3 (15)	
Tongue deviation					0.827
No	17 (85)	18 (90)	19 (95)	19 (95)	
Yes	3 (15)	2 (10)	1 (5)	1 (5)	

## Discussion

After a first transient ischaemic attack or small stroke, the risk of ischaemic stroke is predicted to be 5% within the next 48 h and 10% within the next 14 days. The risk is much greater with individuals who have had ischaemia from large artery atherosclerosis, which accounts for 11% within 48 hours and 24% within 14 days. Therefore, early administration of dual antiplatelet treatment with aspirin and clopidogrel appears to be particularly beneficial in lowering the risk of early stroke recurrence^13^. Clopidogrel was authorized to be utilized in the United States by the Food and Drug Administration (FDA) in November 1997^14^. The combination of aspirin and clopidogrel has been shown to decrease the chance of occlusive vascular events and hence the perioperative stroke risk. Furthermore, antiplatelet medication minimizes the incidence of coronary artery events during or after carotid surgery, especially for those undergoing CEA^15^. A randomized clinical study of 100 patients (all on aspirin) who underwent CEA for symptomatic carotid artery stenosis and were randomly assigned to take clopidogrel or a placebo the night before surgery was conducted. Clopidogrel-treated patients had a 10-fold lower probability of having transcranial detected cerebral emboli in the postoperative period^16^. However, some studies have shown that clopidogrel-treated patients had an increased risk of postoperative cervical haematoma, which is a serious complication of CEA generally occurring in the early stages of the surgery^17,18^. With more intense regimens, the risk of bleeding may increase significantly^15^.

Despite the fact that dual and single antiplatelet treatments are commonly used in CEA, their effectiveness and safety are debatable^3^. Multiple studies have been conducted with inconsistent results regarding the reported increased bleeding risk linked to dual antiplatelet treatment^19^. In a retrospective comparison assessment of CEA patients, Hale *et al*.^20^ observed that dual antiplatelet treatment was independently related to five-fold higher risks of postoperative bleeding or haematoma than single antiplatelet treatment. Dual antiplatelet therapy raises the risk of neck haematoma and necessitates reoperation for an enlarging neck haematoma or significant bleeding from the incision^5,21^. Post-CEA neck haematoma has been associated with higher operational mortality, adverse cardiac events, neurological problems, and cranial nerve damage^5,21^.

Wait *et al*.^22^ discovered a significant rise in postoperative bleeding, cervical swelling, and length of hospital stay in patients who received clopidogrel within 5 days of CEA (*n*=42). According to a recent comprehensive meta-analysis of 36 881 carotid endarterectomies, neck haematoma occurred in 6.77% of patients in the single antiplatelet group and 8.19% of patients in the dual antiplatelet group^15^. In contrast, a multi-institutional study showed that preoperative clopidogrel, whether taken alone or with aspirin, was not related to increased significant bleeding problems in a wide range of peripheral vascular surgeries^19^. Weem *et al*.^23^ presented a meta-analysis comparing single and dual antiplatelet treatment during vascular operations. They revealed no statistically significant differences in restenosis and bleeding.

Little information is available on the clinical outcomes of generic and brand clopidogrel. Only a few studies have examined the pharmacokinetic and pharmacodynamic characteristics of generic versus brand-name clopidogrel^9^. Clopidogrel is available in a variety of salt formulations. The specific salt composition of a product affects its pharmacokinetic and pharmacodynamic characteristics, both of which influence the amount to which it is absorbed, distributed, and removed by the body. Modifying a drug’s salt formulation may thereby modify its physicochemical qualities, affecting its clinical effectiveness and safety^2^. A study on the evaluation of purity in 19 drugs containing clopidogrel, in which there were 18 copies of clopidogrel bisulfate versus the brand name (Plavix), revealed that most of the preparations were not of equal quality compared to the brand drug product; particularly, the portion of impurities was higher, the content of clopidogrel was lower, and the dissolution characteristics were different in the generic formulations^24^.

Because generic and brand drugs differ in terms of peripheral characteristics, such as pill form, colour, inactive binders and fillers, and manufacturing process, having two medications that contain the same active ingredient does not always imply that they will have the same clinical efficacy and safety on the patient^25^. Brosiczky *et al*.^26^ discovered no statistically significant difference in the antiplatelet functional response of clopidogrel besylate versus clopidogrel hydrogen sulfate. In contrast, Neubauer and colleagues. found variability in pharmacodynamics among clopidogrel formulations. It was a crossover study on a group of healthy volunteers that examined two distinct salts of clopidogrel: hydrogen sulfate and besylate. The hydrogen sulfate salt had a higher antiplatelet impact on subjects than the besylate salt. These findings revealed clopidogrel’s substantial inter-individual and intra-individual variability^27^. As with the previous studies, the current study’s findings demonstrated inter-individual and intra-individual diversity in the antiplatelet safety of four distinct clopidogrel formulations. The combination of aspirin and brand clopidogrel (Plavix) and generic clopidogrel (Piax) is linked with greater clinical safety than other generic clopidogrel forms.

Despite the current study having obvious points of strength, being an randomized controlled trial study, we acknowledge that it also has several limitations that need to be addressed. First, it involved only a single centre. Second, the study has a small sample size of 20 patients per each group.

In conclusion, the current study’s findings revealed differences in the clinical safety of four different clopidogrel products administered perioperatively in patients undergoing CEA. More research is needed to further corroborate these findings.

## Ethics approval and consent to participate

The manuscript approved by ethical committee of the University of Sulaimani.

## Consent

Written informed consent was obtained from all participants or the parents in the case that the participants were underage.

## Patient consent for publication

Not applicable.

## Source of funding

No funding was received.

## Conflicts of interest disclosure

The authors declare that they have no competing interests.
